# The VTI1A-TCF4 colon cancer fusion protein is a dominant negative regulator of Wnt signaling and is transcriptionally regulated by intestinal homeodomain factor CDX2

**DOI:** 10.1371/journal.pone.0200215

**Published:** 2018-07-05

**Authors:** Johanne Davidsen, Sylvester Larsen, Mehmet Coskun, Ismail Gögenur, Katja Dahlgaard, Eric Paul Bennett, Jesper Thorvald Troelsen

**Affiliations:** 1 Department of Science and Environment, Roskilde University, Roskilde, Denmark; 2 Department of Surgery, Zealand University Hospital, Roskilde, Denmark; 3 Department of Clinical Immunology, Naestved Hospital, Naestved, Denmark; 4 Department of Gastroenterology, Herlev Hospital, Herlev, Denmark; 5 Copenhagen Center for Glycomics, Department of Odontology, Faculty of Health Sciences, University of Copenhagen, Copenhagen, Denmark; National Cancer Center, JAPAN

## Abstract

Sequencing of primary colorectal tumors has identified a gene fusion in approximately 3% of colorectal cancer patients of the *VTI1A* and *TCF7L2* genes, encoding a VTI1A-TCF4 fusion protein containing a truncated TCF4. As dysregulation of the Wnt signaling pathway is associated with colorectal cancer development and progression, the functional properties and transcriptional regulation of the VTI1A-TCF4 fusion protein may also play a role in these processes. Functional characteristics of the VTI1A-TCF4 fusion protein in Wnt signaling were analyzed in NCI-H508 and LS174T colon cancer cell lines. The NCI-H508 cell line, containing the *VTI1A*-*TCF7L2* fusion gene, showed no active Wnt signaling, and overexpression of the VTI1A-TCF4 fusion protein in LS174T cells along with a Wnt signaling luciferase reporter plasmid showed inhibition of activity. The transcriptional regulation of the *VTI1A-TCF4* fusion gene was investigated in LS174T cells where the activity of the *VTI1A* promoter was compared to that of the *TCF7L2* promoter, and the transcription factor CDX2 was analyzed for gene regulatory activity of the *VTI1A* promoter through luciferase reporter gene assay using colon cancer cell lines as a model. Transfection of LS174T cells showed that the *VTI1A* promoter is highly active compared to the *TCF7L2* promoter, and that CDX2 activates transcription of *VTI1A*. These results suggest that the VTI1A-TCF4 fusion protein is a dominant negative regulator of the Wnt signaling pathway, and that transcription of *VTI1A* is activated by CDX2.

## Introduction

Colorectal cancer is one of the most commonly diagnosed types of cancer in the western world and a leading cause of cancer-related death. The mechanisms behind the development of sporadic colorectal cancer are, despite considerable research, not fully understood [1,2]. Yet, disruptions of the canonical Wnt signaling pathway are known to play a major role in cancer initiation as well as progression and it is estimated that 80–90% of all colorectal cancer tumors harbor mutant Adenomatous Polyposis Coli (APC), an essential scaffold protein in the Wnt signaling pathway [1,3,4].

The central signal transducer in the canonical Wnt signaling pathway is β-catenin. In the absence of Wnt glycoprotein ligands, β-catenin is phosphorylated and subsequently degraded in proteasomes. The interaction between the GSK3 and CK1α kinases and β-catenin is facilitated by the scaffold proteins APC and axin, and combined, the kinases and the scaffold proteins form the degradation complex [5]. Upon binding of secreted Wnt glycoprotein to transmembrane co-receptors, the canonical Wnt signaling pathway is activated which leads to accumulation of intracellular β-catenin. β-catenin then enters the nucleus where it associates with members of the T-cell factor/Lymphoid enhancer-binding factor (TCF/LEF) family of transcription factors and activates transcription of Wnt target genes by displacing the Groucho co-repressor bound to TCF/LEF proteins. If APC or other members of the degradation complex have loss-of-function mutations, β-catenin will not be degraded, resulting in a constitutively active Wnt signaling pathway [1,5,6]. The main binding partner of β-catenin in the colon is TCF4, and studies have shown that TCF4 plays an important part in maintaining the proliferative cells in the colonic crypts, and that natural downregulation of TCF4 expression in colonic epithelial cells migrating up the crypt may induce differentiation [6–8].

Dysregulation of members of the TCF/LEF family of transcription factors has been observed in both colon cancer cell lines, and colon cancer tumors [1,9], and there is indication that the TCF/LEF family of transcription factors work in distinct and opposing roles to maintain the equilibrium between epithelial cell proliferation and terminal differentiation in normal colonic epithelium. LEF1 is specifically expressed in early stages of B-cell differentiation but has also been shown to be expressed in colon cancer tumors [3,10,11]. TCF1 expression is largely restricted to T-lymphocytes in adult tissue but expression has also been detected in colorectal cancer cell lines [12]. In adult mice with a dominant mutated *APC* gene, conditional knockout of TCF4 significantly enhances colon tumor formation, indicating that TCF4 is a tumor suppressor [3]. However, the expression of TCF4 in colon tumors has been shown to correlate to lower survival, indicating oncogenic properties of TCF4 [1]. Thus, the role of TCF4 in colon cancer is not yet fully understood and it may function as both a tumor suppressor and an oncogene [1,3]. With disruptions in the Wnt signaling pathway found in the majority of colon cancer tumors it is nonetheless to be expected that dysregulation of TCF4 has a part to play in colon cancer initiation and/or progression.

Through genomic sequencing, Bass et al. (2011) has identified a recurrent fusion of the *Vps-ten-interacting-1a* (*VTI1A)* and the *T-cell factor 7-like 2* (*TCF7L2)* genes in 3% of primary colorectal adenocarcinomas. The fusion fuses the first three exons of the *VTI1A* gene with the fourth exon of the neighboring gene, *TCF7L2*, thereby omitting the first three exons of the *TCF7L2* gene. The *VTI1A* gene encodes a v-SNARE protein that mediates vesicle transport from late endosomes to the trans-Golgi network, and the *TCF7L2* gene encodes the TCF4 transcription factor. The fusion of the *VTI1A* and *TCF7L2* genes results in a fusion protein, VTI1A-TCF4, in which the N-terminal β-catenin binding domain of TCF4 is lacking, while it still contains the DNA binding domain and the transcription repression domain [13].

Nome et al. (2014) has also detected the VTI1A-TCF7L2 fusion transcript in colorectal cancer tissue samples showing that the genomic rearrangement is functional. They have further shown that 42% of colorectal cancers express the fusion transcript and have detected seven different splice variants of the transcript. The fusion transcripts were also detected in 28% of normal colonic mucosal samples and 5 normal tissue samples from different anatomical sites of the body. The fact that fusions involving *TCF7L2* are highly detectable in colorectal cancers, normal colonic tissue and other normal tissue types, may indicate that these fusions are neither specific to cancer nor to the colon or rectum [14]. However, the similar fusion transcripts observed in individual cancer cell lines induced by genomic rearrangements may still have oncogenic potential. In this study we investigate the transcript generated by the deletion in the NCI-H508 colon cancer cell line, a cell line harboring the gene fusion [13].

The *VTI1A* promoter most likely regulates the transcription of the VTI1A-TCF4 fusion protein. ChIP-seq data indicates that the *VTI1A* promoter contains binding sites for caudal type homeobox 2 (CDX2) [15,16], which is expressed in differentiating cells of the colonic crypts [17,18], and not in the proliferating cells, where TCF4 is expressed [8]. This means that the VTI1A-TCF4 fusion protein may be expressed throughout the entire colonic crypt and not only at the base where expression of TCF4 is normally seen. Further, research indicates that dysregulation of CDX2 in the colon plays a role in the development and progression of colorectal cancer [19–21].

The VTI1A-TCF4 fusion protein has been shown to play a critical role in anchorage-independent growth of the colon cancer cell line NCI-H508, indicating that the VTI1A-TCF4 protein has functional properties [13]. Other members of the TCF/LEF family of transcription factors naturally occur as truncated isoforms with no β-catenin binding domains, creating proteins with dominant negative properties [22]. The functional properties of the VTI1A-TCF4 fusion protein have not yet been investigated, but research suggests that it might also exhibit dominant negative properties as it also lacks the β-catenin binding domains [23,24]. The aim of this study was to determine the functional properties of the VTI1A-TCF4 fusion protein and to investigate the level of transcriptional activity from the *VTI1A-TCF7L2* fusion gene in colon cancer cells compared to that of wildtype *TCF4*. Additionally, it aims to explore the role of CDX2 in regulation of transcription of the *VTI1A-TCF7L2* fusion gene.

## Materials and methods

### Cloning of *VTI1A* and *TCF7L2* promoter-reporter constructs

The *VTI1A* promoter region (735 bp, from position -765 to -30, NM_145206) was amplified from human genomic DNA using the VTI1A-735 primer set and cloned with the In-Fusion HD cloning system (Clontech, #639649) into the *HindIII* site of the modified firefly luciferase reporter pGL4.10 vector, resulting in the pGL4.10-VTI1A-735 construct. Plasmids for deletion analysis were created using the same reverse primer but different forward primers. The promoter region for *TCF7L2* (1405 bp, from position -1545 to -140, NM_030756) was amplified from human genomic DNA using the TCF7L2-1405 primer set and cloned into the pGL4.10 vector using the *Xho1* and *HindIII* sites, resulting in the pGL4.10-TCF7L2-1405 construct. The plasmids were sequence verified (Beckman Coulter Genomics). Primer sequences can be found in S1 Table.

### Cloning of VTI1A-TCF4 expression plasmid

NCI-H508 cells were seeded at 500.000 cells per well in a 6-well plate and incubated at 37°C and 5% CO_2_ for 48 hours. The cells were harvested and total RNA was purified with Total RNA Kit I (Omega Bio-tek, #R5834-02). RNA to cDNA synthesis was performed using First Strand cDNA Synthesis kit (Thermo Scientific, #K1612) according to manufactures protocol. The cDNA was amplified with VTI1A-TCF4 sequence specific primers and gel-purified *VTI1A-TCF7L2* sequence was cloned into *NheI* digested pLVX-CherryPicker2 vector (Clontech, #632581) using the In-Fusion cloning system (Clontech, #639649) resulting in the VTI1A-TCF4 fusion protein expression plasmid. The plasmid was sequence verified (Beckman Coulter Genomics). Primer sequences can be found in S1 Table. Protein expression from the VTI1A-TCF4 expression plasmid was verified by Western Blot, see S1 Fig. LS174T cells were seeded at 600.000 cells per well in a 6-well plate with or without 1.2 μg VTI1A-TCF4 expression plasmid, according to transfection protocol below, and incubated at 37°C and 5% CO_2_ for 48 hours. The cells were then lysed in ice cold 1XLDS buffer in RIPA buffer. Each sample was heated to 82°C for 3 minutes and separated on 4–12% BisTris SDS PAGE with 1XMOPS SDS running buffer. Proteins were transferred to PVDF membrane 0.45 μm (Invitrolon), and the membrane was incubated with TCF4/TCF7L2 (C48H11) Rabbit mAb (Cell Signaling) 1:1000 overnight at 4°C and subsequently incubated for 2 hours with Pierce 332260 Goat Anti-Rabbit IgG, HRP-linked Antibody 1:5000 followed by detection by Pierce’s Dura detection reagent.

### Cell culture

LS174T, NCI-H508, and Caco-2 cell lines were used in this study. LS174T and Caco-2 cells were grown in DMEM 4.5 g/L Glucose with UltraGlutamine (Lonza, #BE12-604F-U1) containing 10% heat-inactivated Fetal Bovine Serum and 100 U/ml penicillin and streptomycin. The NCI-H508 cells were cultured in RMPI 1640 medium with L-Glutamine (Lonza, #BE12-702F) added 10% Fetal Bovine Serum and 100 U/ml penicillin and streptomycin. All cells were grown at 37°C and 5% CO_2_ in T75 flasks and passaged twice a week. An LS174T cell line with both *CDX2* alleles genetically knocked out, obtained from Pinto et al. (2017), was also used. These cells were cultured as the wild type LS174T cell line.

### Transfection

For transfections, LS174T cells and NCI-H508 cells were seeded at 100.000 cells per well in 24-well plates. Caco-2 cells were seeded at 50.000 cells per well. Cell transfections were done using 2 μM Polyethyleneimine (Alfa Aesar #43896). Four replicas were made and each transfection was carried out at least twice. The luciferase reporter plasmid pGL4.10-VTI1A was used for the transfections, and the pCMV-lacZ plasmid was used to determine transfection efficiency control. The TOPFlash and FOPFlash reporter system was used to determine Wnt signaling [25]. For co-transfections, plasmids expressing CDX2, or the VTI1A-TCF7L2 fusion protein were added the transfection. To normalize to a total amount of 0.3 μg DNA per well pBluescript SK+ plasmid was added. 48 hours after transfection the cells were harvested, and the luciferase and β-galactosidase activities were analyzed using the Dual Light assay kit (Invitrogen, #T1004) on a GloMax 96 Microplate Luminometer with dual injectors (Promega). The activity for both luciferase and β-galactosidase were measured for 5 seconds, and each luciferase activity was normalized to β-galactosidase activity. The activity is stated in relative fluorescence units (RFU).

### Chromatin immunoprecipitation

Confluent LS174T cells were cross-linked by formaldehyde treatment and then sonicated. Immunoprecipitation with CDX2 and HA was done as described in Coskun et al. 2012 [26]. Verification of immunoprecipitation enrichment was performed with quantitative PCR (qPCR) using a Stratagene MX3005P (Agilent Technologies) cycler with Quantitect SYBR Green PCR Mastermix (Qiagen, #204141) according to manufactures protocol. The primer set CDX2 ChIP was used and can be found in S1 Table. Quantification of the ChIP-DNA was done using the delta-delta method described by Livak and Schmittgen, 2001 [27].

### Statistical methods

Values are presented as means with standard deviations, and groups were compared using two-sided Student’s t-test. P-values lower than 0.05 were considered significant. * indicates p<0.05, ** indicates p<0.01, *** indicates p<0.001, **** indicates p<0.0001.

## Results

### The VTI1A-TCF4 fusion protein has dominant negative properties

**Fig 1:**
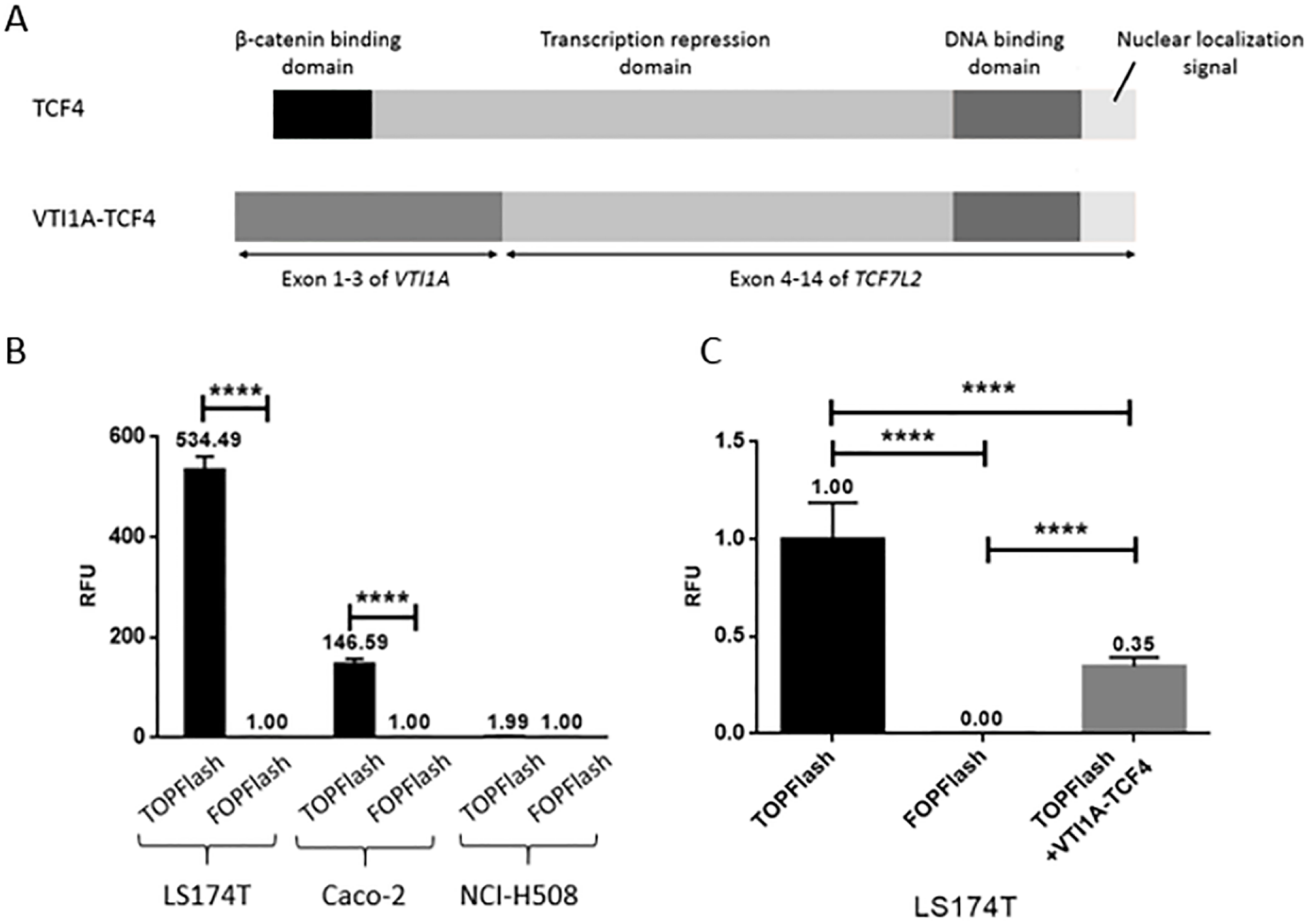
Wnt signaling in colon cancer cell lines. ** a** Wildtype TCF4 contains a β-catenin binding domain, a transcription repression domain, a DNA binding domain, and a nuclear localization signal. With the fusion with VTI1A the resulting fusion protein, VTI1A-TCF4, contains the first three exons of VTI1A and lacks the β-catenin binding domain of TCF4. The rest of the TCF4 domains are still present. **b** The cells lines LS174T, Caco-2 and NCI-H508 were transfected with the TOPFlash and FOPFlash reporter plasmids, and luciferase activity was measured. Activity is stated in mean values relative to FOPFlash activity for each cell line and corrected with β-galactosidase activity. Error bars indicate SD, **** indicates p<0.0001, n = 4 **c** The LS174T cell line, showing high Wnt activity, was transfected with the TOPFlash and co-transfected with a plasmid expressing the VTI1A-TCF4 fusion protein. Activity is stated in mean values relative to TOPFlash activity and corrected with β-galactosidase activity. Error bars indicate SD, **** indicates p<0.0001, n = 8.

The level of Wnt signaling in LS174T, NCI-H508, and Caco-2 cell lines was investigated using the TOPFlash/FOPFlash reporter system [25]. The TOPFlash reporter plasmid contains seven TCF binding sites upstream a luciferase reporter gene that will be transcribed during active Wnt signaling. The FOPFlash reporter plasmid works as a negative control and contains mutated TCF binding sites upstream a luciferase reporter gene and can therefore not be activated by binding of TCF4 during Wnt signaling [25]. Results showed that the transcriptional activity of the TOPFlash reporter plasmid in LS174T cells was more than 500-fold higher compared to the activity of the FOPFlash reporter plasmid (p<0.0001, Fig 1b). A ~150-fold higher TOPFlash transcriptional activity was seen in Caco-2 cells compared to FOPFlash activity (p<0.0001). Both cell lines have loss-of-function mutations in regulators of the Wnt signaling pathway. The LS174T cell line has mutated *β-catenin*, making CK1α unable to bind [28], and the Caco-2 cell line is APC mutated [29]. These mutations result in constitutively active Wnt signaling, supporting the high level of TOPFlash activation. When transfecting NCI-H508 cells, that carry the *VTI1A*-*TCF7L2* gene fusion (Fig 1a) [13], with the TOPFlash reporter plasmid, the luciferase activity was not observed to be significantly higher than that of the FOPFlash reporter plasmid (Fig 1b). These results suggest that either the NCI-H508 cell line does not have active Wnt signaling, or the VTI1A-TCF4 fusion protein in the NCI-H508 cells is not able to activate transcription from the TCF binding sites.

To determine whether the absence of TOPFlash activation in NCI-H508 cell was a result of the properties of the VTI1A-TCF4 fusion protein, LS174T cells were co-transfected with a plasmid expressing the VTI1A-TCF4 fusion protein and the TOPFlash plasmid. A 3-fold decrease in luciferase activity was observed when co-transfecting with VTI1A-TCF4 fusion protein expression plasmid (p<0.0001, Fig 1c). This shows that the presence of the VTI1A-TCF4 fusion protein in the LS174T cells, which has two functional *TCF7L2* alleles, has the ability to significantly reduce the strong Wnt signaling in LS174T cells, and indicates that the VTI1A-TCF4 fusion protein has dominant negative properties.

### The *VTI1A* promoter is highly active compared to the *TCF7L2* promoter in LS174T cells

**Fig 2:**
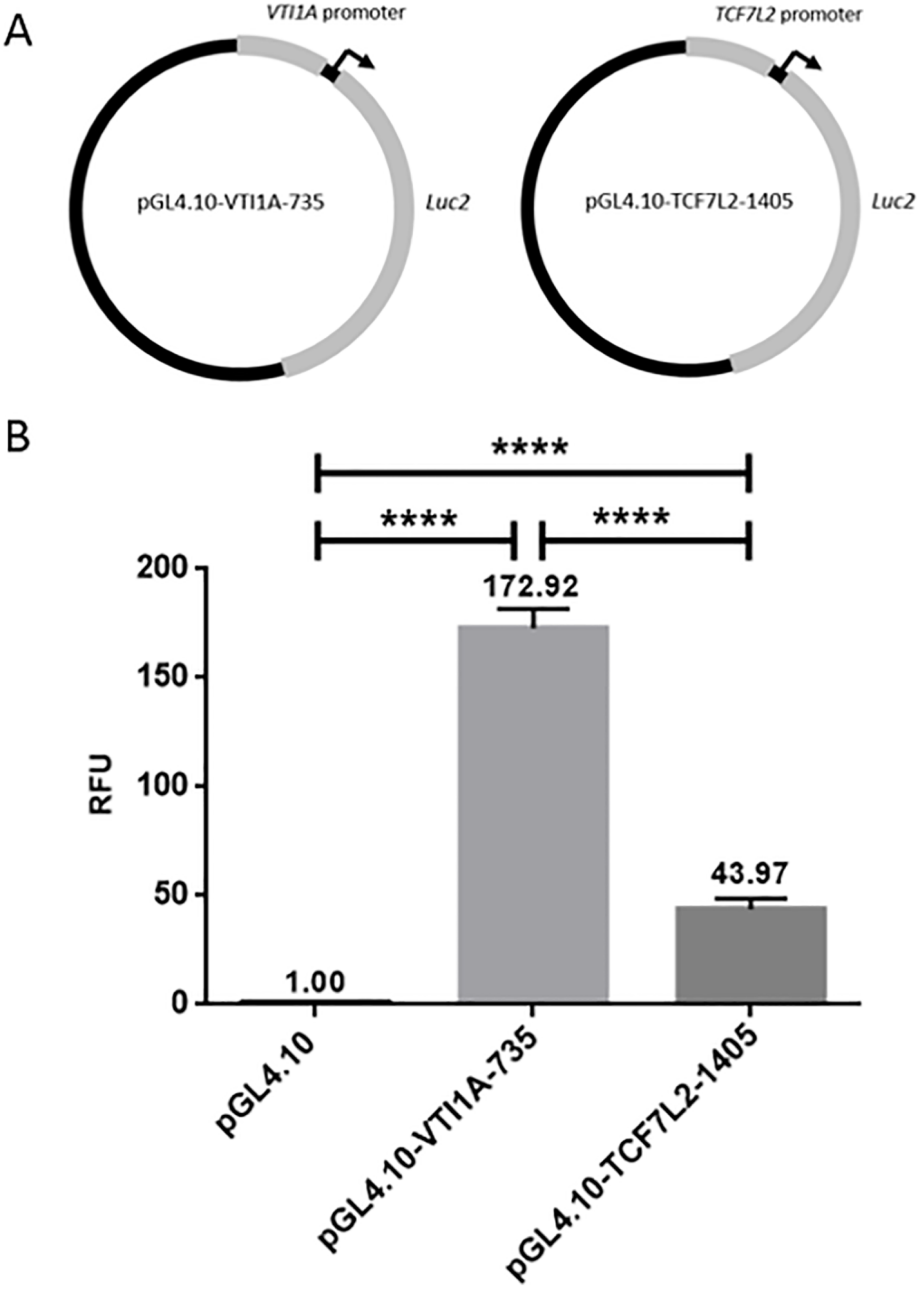
Transcriptional activity of the VTI1A and TCF7L2 promoters. ** a** The pGL4.10-VTI1A-735 and pGL4.10-TCF7L2-1405 plasmids were created by inserting the promoter regions of VTI1A and TCF7L2, respectively, upstream the luciferase gene in the pGL4.10 plasmid. **b** LS174T cells were transfected with either the pGL4.10-VTI1A-375 or the pGL4.10-TCF7L2-1405 reporter plasmids. Activity is stated in mean values relative to pGL4.10 activity and corrected with β-galactosidase activity. Error bars indicate SD, **** indicates p<0.0001, n = 8.

In order to investigate promoter activity of *VTI1A* and *TCF7L2*, reporter plasmid constructs containing the *VTI1A* promoter (pGL4.10-VTI1A-735) and the *TCF7L2* promoter (pGL4.10- TCF7L2-1405) were generated. These two constructs (Fig 2a) were transfected into the LS174T cell line. The luciferase activity of pGL4.10-VTI1A-735 was significantly higher, ~170-fold, compared with the activity of the pGL4.10 empty vector (p<0.0001) showing that the promoter is transcriptionally active in LS174T cells (Fig 2b). The activity of pGL4.10-TCF7L2-1405 was likewise significantly higher than pGL4.10 with a >40-fold increase in luciferase activity (p<0.0001). The luciferase activity of the construct containing the *VTI1A* promoter, pGL4.10-VTI1A-735, was significantly higher (p<0.0001) than the *TCF7L2* promoter construct. This shows that the promoter region for the *VTI1A-TCF7L2* fusion gene, encoding the VTI1A-TCF4 fusion protein, is much more transcriptionally active than the promoter for *TCF7L2* in the LS174T cell line. This will most likely result in altered expression of VTI1A-TCF4 compared to TCF7L2 *in vivo*.

### The transcriptional activity of the *VTI1A* promoter is regulated by CDX2 in cultured LS174T cells

**Fig 3:**
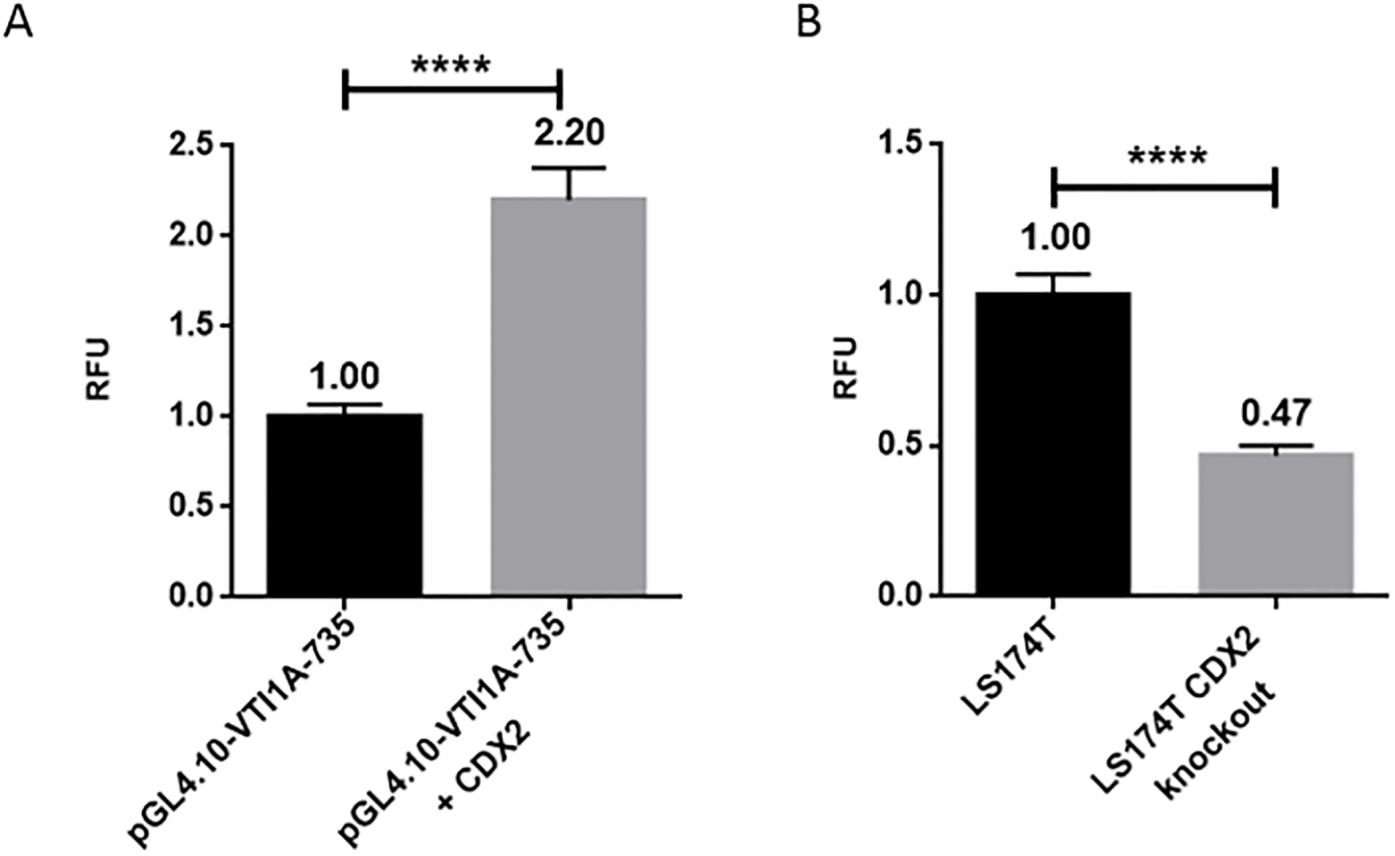
Transcriptional activity of CDX2. ** a** LS174T cells were transfected with the pGL4.10-VTI1A plasmid-735 and co-transfected with a CDX2 expression plasmid. Activity is stated in mean values relative to pGL4.10 activity and corrected with β-galactosidase activity. Error bars indicate SD, **** indicates p<0.0001, n = 4. **b** The LS174T wild type cell line and a LS174T CDX2 knockout cell line was transfected with the pGL4.10-VTI1A plasmid. Activity is stated in mean values relative to pGL4.10 activity and corrected with β-galactosidase activity. Error bars indicate SD, **** indicates p<0.0001, n = 4.

Genome-wide CDX2 ChIP-seq data from LS174T cells [30], shows a CDX2 ChIP-seq peak in the *VTI1A* promoter region, and the JASPAR CORE database identifies two potential CDX2 binding sites within this peak [31]. To investigate the relevance of CDX2’s role in the transcriptional regulation of the *VTI1A* promoter region, a CDX2 expression plasmid and the pGL4.10-VTI1A-735 reporter plasmid were co-transfected into LS174T cells. Overexpression of CDX2 results in a 2-fold increase (p<0.0001) in transcriptional activity of pGL4.10-VTI1A-735 (Fig 3a). These results indicate that CDX2 has an up-regulatory effect on the transcriptional activity of the *VTI1A* promoter. Further indication of CDX2 regulation on the transcriptional activity of the *VTI1A* promoter was established in a LS174T *CDX2* knockout cell line from Pinto et al. (2017). When transfecting the cells with the pGL4.10-VTI1A-735 reporter plasmid, the luciferase activity in the *CDX2* knockout cells decreased by 50% compared to the activity of the plasmid in the LS174T wildtype cells (Fig 3b).

**Fig 4:**
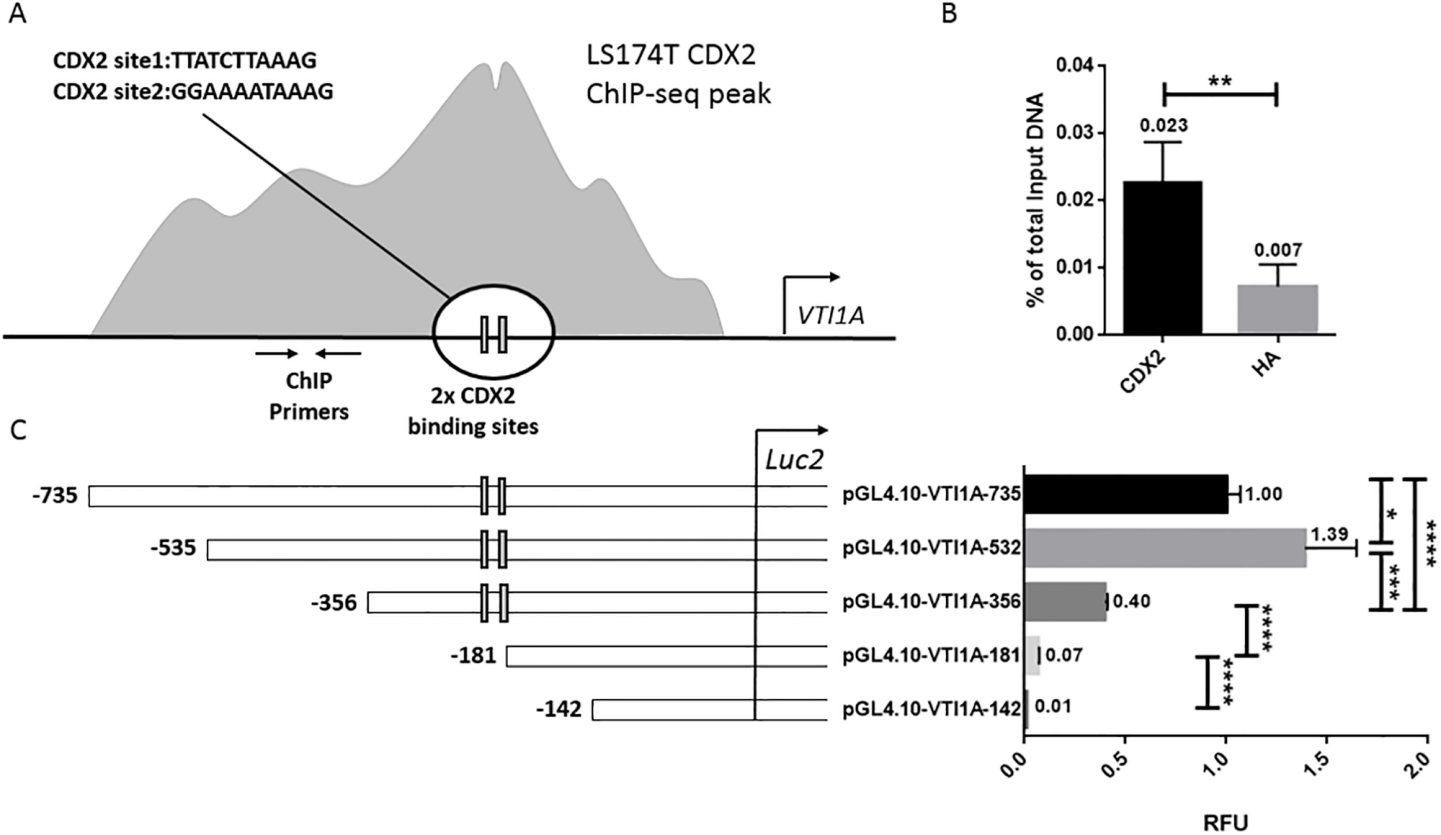
CDX2 regulation of *VTI1A* transcriptional activity. **a** LS174T CDX2 ChIP-seq data was used to identify a CDX2 peak in the VTI1A promoter region [30], and the JASPAR CORE database identifies two potential binding sites within this region [31]. **b** qPCR was performed on CDX2 immunoprecipitated DNA from LS174T cells with primers amplifying the 100 bp region seen in fig 4A. Error bars indicates SD, ** indicates p<0.01, n = 4 **c** Plasmids for deletion analysis with different lengths of the VTI1A promoter inserted in the pGl4.10 plasmid. The two possible CDX2 binding sites are marked. The deletion analysis was carried out in LS174T cells and the luciferase activity is stated in mean values relative to the pGL4.10-VTI1A-735 plasmid and is corrected with β-galactosidase activity. Error bars indicate SD, * indicates p<0.05, *** indicates p<0.001, **** indicates p<0.0001, n = 4.

To further elaborate on CDX2’s role in the transcriptional regulation of the *VTI1A* promoter region, and thereby the expressional regulation of the VTI1A-TCF4 fusion protein, CDX2 ChIP assay on LS174T cells was performed, where chromatin-protein complexes were immunoprecipitated with CDX2-specific antibody or HA antibody, used as a negative control. The region investigated was determined from the CDX2 peak in the ChIP-seq data from Pinto et al. 2017, and can be seen on Fig 4a. The amount of CDX2 immunoprecipitated DNA was measured by qPCR using *VTI1A* promoter-specific primers flanking the potential binding region. The DNA was fragmented to a size of 300–400 bp in the ChIP assay. Thus, sequences flanking the CDX2 binding sites will also be precipitated. A good primer set for amplification of sequences nearby the ChIP-seq peak was chosen. Results show a significant enrichment of CDX2 ChIP DNA compared to the negative HA antibody control (p<0.01, Fig 4b). These results indicate that the CDX2 ChIP-seq peak in the *VTI1A* promoter contains CDX2 binding sites.

The validity of the identified CDX2 binding site as well as the two potential binding sites found using the JASPAR CORE database [31] was investigated through a deletion analysis using different lengths of the *VTI1A* promoter region cloned into the pGL4.10 plasmid (Fig 4c). The results of the deletion analysis show a 1.4-fold higher luciferase activity of the pGL4.10-VTI1A-532 plasmid compared to the activity of the pGL4.10-VTI1A-735 plasmid (Fig 4c). This increase in activity might be a result of inhibitory regions in the pGL4.10-VTI1A-735 plasmid. The luciferase activity of the pGL4.10-VTI1A-356 plasmid is 3.5-fold lower compared to the activity of the pGL4.10-VTI1A-532 plasmid, possibly an effect of this plasmid not containing the CDX2 regulatory region found previously. When comparing the luciferase activity of the pGL4.10-VTI1A-356 with that of the pGL4.10-VTI1A-181 plasmid a 5.7-fold decrease in activity and an almost complete abrogation of luciferase activity can be seen, indicating that fragment of *VTI1A* promoter omitted in the pGL4.10-VTI1A-181 plasmid is essential for the regulatory effect of CDX2 on the promoter. Furthermore, the CDX2 binding sites identified by the JASPER database are located in this fragment of the promoter region.

## Discussion

Some members of the TCF/LEF family of transcription factors naturally occur as N-terminally truncated isoforms lacking the β-catenin binding site [22]. The nuclear localization signal, the DNA binding domain, and the transcription repression domain are still present in the truncated forms. This means that truncated isoforms of TCF/LEF are able to localize to the nucleus, bind to DNA binding sites, and recruit the Groucho co-repressor to the transcription repression domain. As the β-catenin binding site is not present in these truncated variants of TCF/LEF, β-catenin is unable to displace Groucho, resulting in repression of transcription [9,32]. Studies by Goncalves et al. and Van der Wetering et al. shows that constructs of truncated TCF4, that lack the N-terminal containing the β-catenin binding site, functions as dominant negative regulators of endogenous β-catenin/TCF complexes [23,24]. Our results show that the VTI1A-TCF4 fusion protein, which also lacks the N-terminal domain with β-catenin binding domain, while it contains the nuclear localization signal, the DNA binding domain, and the transcription repression domain (Fig 1a), has dominant negative effects on WNT signaling.

Bass et al. (2011) did not investigate the regulatory properties of the VTI1A-TCF4 fusion protein, however they propose that it does not act as a full dominant negative protein as engineered dominant-negative *TCF4* alleles have been shown to strongly inhibit proliferation of colorectal carcinoma cell lines [24]. Furthermore, Bass et al. (2011) demonstrated that the VTI1A-TCF4 protein has a critical role in anchorage-independent growth of VTI1A-TCF4 fusion protein positive NCI-H508 cells, which would not have been expected if the VTI1A-TCF4 fusion protein is fully dominant negative [13]. In the study by Van der Wetering et al. (2002), doxycycline inducible plasmids expressing N-terminally truncated TCF4 were transfected into LS174T cells, and upon doxycycline induction the activity of TOPFlash was completely abrogated and the proliferation of the cells was inhibited [24]. However, when expressing the VTI1A-TCF4 fusion protein in LS174T cells, a complete abrogation of TOPFlash activity was not seen. This indicates that the fusion protein might exhibit different functional effects than that of a constructed truncated TCF4 protein and might therefore still allow for cell proliferation. Furthermore, the VTI1A-TCF4 fusion protein contains domains encoded by the *VTI1A* gene, which might add other functionalities to the VTI1A-TCF4 fusion protein.

Our results show that the *VTI1A* promoter region is significantly more active in LS174T cells than the promoter region for *TCF7L2*. This could mean increased expression of the VTI1A-TCF4 fusion protein in the proliferating cells of colonic crypts, where TCF4 is normally present [8]. The *VTI1A* promoter region was also shown to be regulated by CDX2 which is highly expressed in the upper two-thirds of the crypts [17,33]. Our results suggest that the VTI1A-TCF4 fusion protein will be expressed in the upper two-thirds of the crypts as well as at the base of the crypts. Studies have shown that TCF4 plays an important part in maintaining the proliferative cells in the colonic crypts, and that natural down-regulation of TCF4 expression as cells move up the colonic crypts may trigger colonic epithelial cell differentiation [6–8]. *Tcf7l* knockout mice die within 24 hours after birth and show no proliferating crypt stem cell compartments, supporting the idea that TCF4 plays an essential role in maintaining the proliferative compartment in the crypt [34].

**Fig 5:**
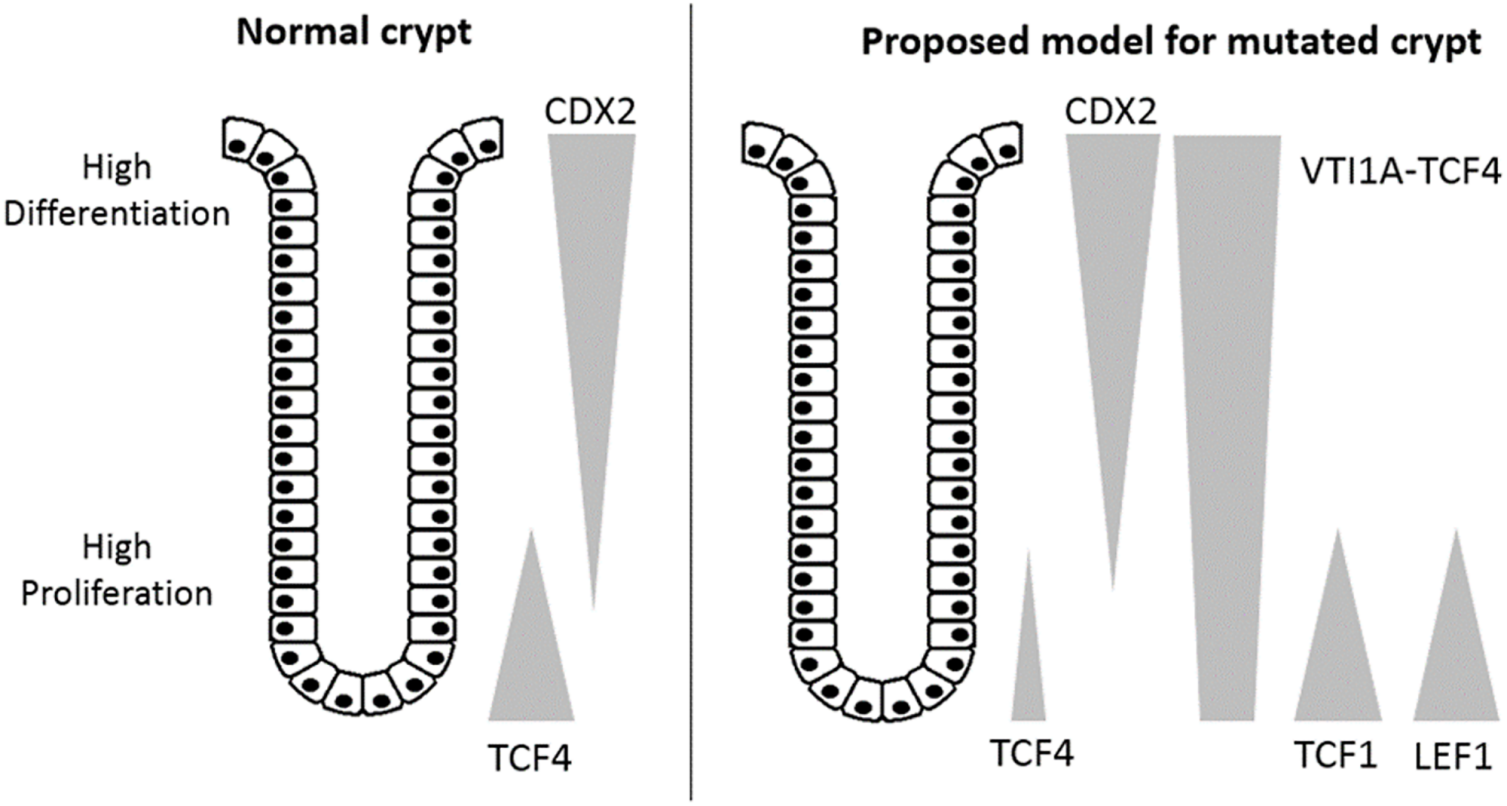
Expression in the colonic crypts. In normal crypts TCF4 is expressed at the base of the crypts [8] and as cells migrate towards the top of the crypts the expression of TCF4 decreases while expression of CDX2 increases [17,33]. In a proposed model for a crypt with cells carrying the *VTI1A*-*TCF7L2* fusion, the VTI1A-TCF4 fusion protein will be expressed throughout the crypt. As β-catenin is not able to bind to VTI1A-TCF4 it may instead bind and activate other members of the TCF/LEF family of transcription factors, e.g. TCF1 and LEF1.

The main binding partner of β-catenin in the colon is TCF4, but colon tumors isolated from *APC* mutated mice have an increased expression of LEF1 and TCF1 [3]. During active Wnt signaling, β-catenin will accumulate in the cell nucleus and with no binding site in the VTI1A-TCF4 fusion protein, β-catenin might bind and activate other members of the TCF/LEF family of transcription factors, such as TCF1 and LEF1. As the TCF/LEF family of transcription factors are indicated to work in distinct and opposing ways to regulate and maintain the equilibrium in cell proliferation and differentiation [1,9], a shift in this balance may contribute to the development or progression of colorectal cancer. A proposed model for the expression pattern of transcription factors in the mutated crypts can be seen in Fig 5.

In conclusion, we have determined that the VTI1A-TCF4 fusion protein transcribed from *VTI1A-TCF7L2* gene fusion has dominant negative properties and the transcription of this protein is activated by CDX2 in cultures LS174T cells. The role of the VTI1A-TCF4 fusion protein in colorectal cancer still needs to be investigated. However, as dysregulation of the Wnt signaling pathway plays an important role in the development of colorectal cancer the shift from wild type TCF4, that can function as a transcriptional activator, to the dominant negative VTI1A-TCF4 in mutated colonic cells will most likely have an effect on colon homeostasis and may perhaps contribute to the development of colorectal cancer.

## Supporting information

S1 Fig Protein expression of VTI1A-TCF4. LS174T cells were transfected with the VTI1A-TCF4 expression plasmid and after 48 hours protein was extracted and used for Western Blot. The blot was incubated with TCF4 antibody. In cells transfected with the VTI1A-TCF4 expression plasmid a clear band can be seen at approx. 43 kDa, corresponding to the length of the VTI1A-TCF4 fusion protein. In the cells not transfected with the VTI1A-TCF4 expression plasmid, this band cannot be seen. For both samples the wild type TCF4 protein is seen at approx. 75 and 55 kDa.
(PDF)

S1 Table Primers. List of primers used in the study.
(DOCX)
