# Supplementary figures and images for: The VTI1A-TCF4 colon cancer fusion protein is a dominant negative regulator of Wnt signaling and is transcriptionally regulated by intestinal homeodomain factor CDX2
Source: PLoS One. 2018 Jul 5;13(7):e0200215. doi: 10.1371/journal.pone.0200215 (PMC6033461; doi:10.1371/journal.pone.0200215)

S1 figure

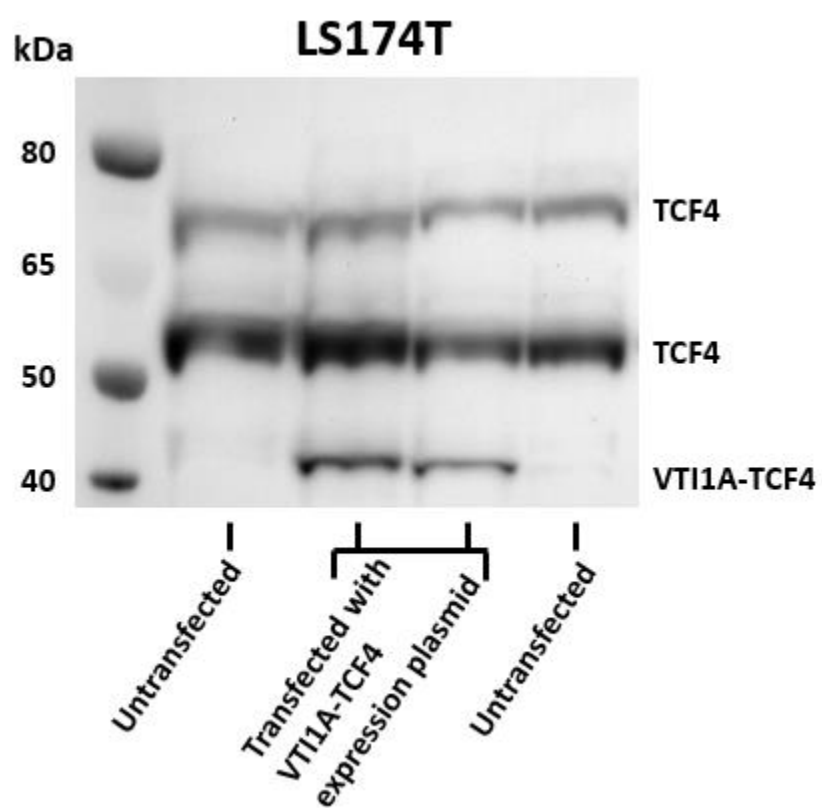

Supplement: S1 Fig — LS174T cells were transfected with the VTI1A-TCF4 expression plasmid and after 48 hours protein was extracted and used for Western Blot. The blot was incubated with TCF4 antibody. In cells transfected with the VTI1A-TCF4 expression plasmid a clear band can be seen at approx. 43 kDa, corresponding to the length of the VTI1A-TCF4 fusion protein. In the cells not transfected with the VTI1A-TCF4 expression plasmid, this band cannot be seen. For both samples the wild type TCF4 protein is seen at approx. 75 and 55 kDa. (PDF) [file pone.0200215.s001.pdf]

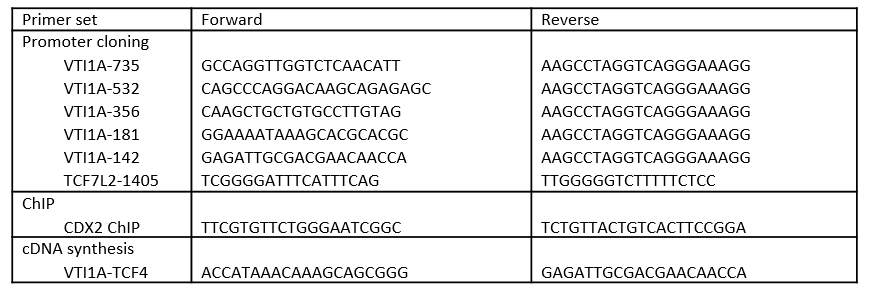
 **Table S1**

Supplement: S1 Table — List of primers used in the study. (DOCX) [file pone.0200215.s002.docx]
